# Data on an intervention to reduce readmissions after open heart valve surgery

**DOI:** 10.1016/j.dib.2019.103926

**Published:** 2019-04-23

**Authors:** Britt Borregaard, Jordi Sanchez Dahl, Lars Peter Schødt Riber, Ola Ekholm, Kirstine Lærum Sibilitz, Marc Weiss, Jan Sørensen, Selina Kikkenborg Berg, Jacob Eifer Møller

**Affiliations:** a Department of Cardiothoracic and Vascular Surgery, Odense University Hospital, Odense, Denmark; b OPEN, Odense Patient Data Explorative Network, Odense University Hospital, Odense, Denmark; c Faculty of Health Sciences, University of Southern Denmark, Odense, Denmark; d Department of Cardiology, Odense University Hospital, Odense, Denmark; e National Institute of Public Health, University of Southern Denmark, Copenhagen, Denmark; f The Heart Centre, Rigshospitalet, Copenhagen University Hospital, Copenhagen, Denmark; g Centre for Health Economics Research (COHERE), National Institute of Public Health, University of Southern Denmark, Odense, Denmark; h Healthcare Outcomes Research Centre (HORC), Royal College of Surgeons in Ireland, Dublin, Ireland; i Department of Clinical Medicine, Faculty of Health and Medical Sciences, University of Copenhagen, Denmark

**Keywords:** Heart valves, Readmission, Propensity matched, Heart valve surgery

## Abstract

Data describe supplementary tables and figures related to the research article; Effect of early, individualised and intensified follow-up after open heart valve surgery on unplanned cardiac hospital readmissions and all-cause mortality [1].

Data on patients undergoing open heart valve surgery were presented in 308 patients in a prospective cohort and compared with 980 patients in a historical cohort. Included figures show inclusion and exclusion of patients (flowchart) and the specific elements of the intervention. Tables show causes of readmission and sensitivity analyses of differences among patients in the prospective intervention group compared with patients in the historical control group. Further results, interpretation and discussion of the included data can be found in the main research paper.

**Table:** Specifications Table

Subject area	*Health Science*
More specific subject area	*Cardiac surgery, Cardiology*
Type of data	*Tables, figures*
How data was acquired	*Prospective cohort study compared with historical control group*
Data format	*Analysed*
Experimental factors	*Effect of an intervention consisting of early, individualised and intensified follow-up after open heart valve surgery*
Experimental features	*Patients undergoing open heart valve surgery were included in a prospective cohort. The intervention was initiated with a risk assessment performed at discharge, resulting in patients considered being at high, intermediate or low risk of readmission. Follow-up after discharge was planned according to the risk assessment and lasted for four weeks.*
Data source location	*Region of Southern Denmark, Denmark*
Data accessibility	*The data are available with this article. The raw data are available from corresponding author after approval by the Danish Data Protection Agency*
Related research article	*Borregaard B, Dahl J, Riber LPS, Ekholm O, Sibilitz KL, Weiss M, Sørensen J, Berg SK, Møller JE. Effect of early, individualised and intensified follow-up after open heart valve surgery on unplanned cardiac hospital readmissions and all-cause mortality. Int J Cardiol 2019. 2019/04/23*

**Table:** 

**Value of the data** • Data presented in the current paper provide information about consecutive patients undergoing open heart valve surgery, including a prospective cohort enrolled in an intervention programme aiming to reduce readmissions. • Specific elements of the intervention consisting of early, individualised and intensified follow-up are visualised and can be used by clinicians for future outpatient follow-up. • Detailed data on different causes of cardiac readmission, including differences among intervention and control group are provided. Data can help caregivers gain knowledge of readmission patterns after open heart valve surgery. • The provided data can be useful for other multidisciplinary teams, as the effect of the intervention can be implemented and tested in other populations of patients undergoing open heart valve surgery.

## 1 Data

**Fig. 1:**
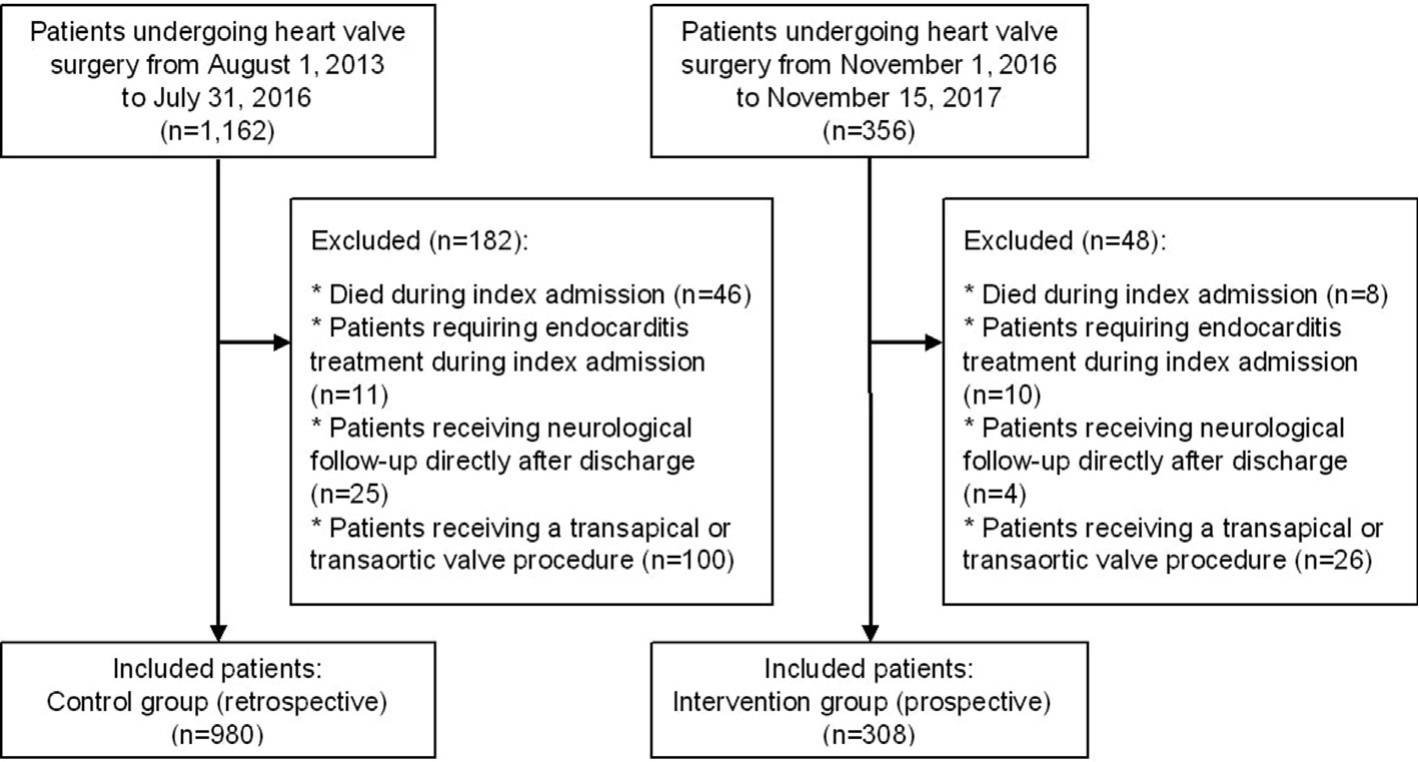
Flowchart of included patients. Flowchart of included patients in the prospective intervention group and the historical control group. The period from August 2016 to October 2016 was excluded while the included health care professionals underwent training of the intervention in clinical practice.

**Fig. 2:**
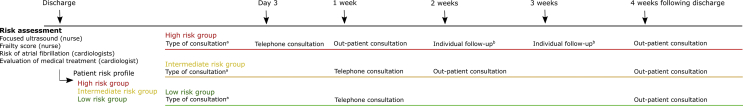
The Intervention; Risk assessment at discharge and planned consultations. Risk assessment and follow-up during the intervention period. a All consultations after discharge were nurse-led with clinical back-up as needed (cardiologist or heart surgeon). b Individual follow-up according to symptoms leading to; no consultation, telephone- or out-patient consultation.

The data shared are supplementary tables and figures of analysed data from the INVOLVE study. In brief, data on a prospective consecutive cohort of patients were compared with data from a historical, control group. Data were based on adult patients undergoing open heart valve surgery at a tertiary hospital in Denmark. Fig. 1 outlines flowchart of included patients in both prospective and historical cohort, and the specific elements of the intervention are visualised in Fig. 2. Causes of readmissions and differences among the prospective cohort and the historical control group are summarised in Table 1. Finally, in Table 2, differences in readmission rates are described, including differences in unplanned and planned cardiac hospital readmissions, all-cause readmissions and mortality among the intervention group and the historical control group.

**Table 1:** Causes of first, unplanned cardiac hospital readmission.

*Causes of readmission, n (%)*	*Of the overall population*		*Of patients readmitted*	
	Intervention (n = 308)	Historical control (n = 980)	Intervention (n = 70)	Historical control (n = 366)
Pericardial effusion	10 (3.2)	68 (6.9)	10 (14.3)	68 (18.6)
Pleura effusion	5 (1.6)	30 (3.1)	5 (7.1)	30 (8.2)
Atrial fibrillation/flutter	15 (4.9)	61 (6.2)	15 (21.4)	61 (16.7)
Heart failure	5 (1.6)	18 (1.8)	5 (7.1)	18 (4.9)
Infections, all^a^	18 (5.8)	82 (8.4)	18 (25.7)	82 (22.4)
Cardiac symptoms without other specific cause^b^	5 (1.6)	37 (3.8)	5 (7.1)	37 (10.1)
Others, presumed to be related to the surgery^c^	12 (3.9)	70 (7.1)	12 (17.1)	70 (19.1)

Due to fewer than three cases in several groups, causes were summed.

a Pneumonia, endocarditis, sternal infections and unspecified infections.

b Angina pectoris, dyspnea, vertigo or syncope.

c E.g., Anaemia, dysregulation of anti-coagulation therapy, new pacemaker implantation, stroke, acute coronary syndrome, re-operation, wound problems, gastro-intestinal bleeding, medical problems (not anti-coagulation), ventricular tachycardia.

**Table 2:** Sensitivity analyses, differences among groups.

	Intervention (n = 308)	Historical control (n = 980)	p*
Composite events (Event of first, unplanned readmission or all-cause mortality), n (%)	70 (23)	372 (38)	<0.001*
All-cause mortality, n (%)	5 (1.6)	18 (1.8)	0.805
***Unplanned, cardiac readmission***			
All, first unplanned, cardiac readmissions, n (%)	70 (23)	366 (37)	<0.001*
Readmissions per readmitted patient, mean (SD)	2.3 (0.5)	2.4 (0.9)	0.187
Days readmitted per readmitted patient, mean (SD)	7.1 (9.4)	9.1 (14.5)	0.256
***Planned, cardiac readmission***			
All planned, cardiac readmissions, n (%)	12 (3.9)	47 (4.8)	0.510
***All-cause readmissions***			
All readmissions, n (%)	93 (30.2)	437 (44.6)	<0.001*

Differences in means between the groups were tested using the t-test and differences in proportions between all diagnostic groups were tested with the Pearson χ2-test.

Significance level, p < 0.05.

## 2 Experimental design, materials and methods

### 2.1 Population

To obtain the present data, we included adult patients undergoing heart valve surgery at Odense University Hospital, Denmark, within two different time periods. Patients included in the intervention period were consecutively enrolled in a prospective cohort from November 2016 to November 2017. Patients undergoing heart valve surgery at the same hospital during the period from August 2013 to July 2016 comprised the historical control group. Exclusion criteria were; patients living outside the Region of Southern Denmark, patients undergoing transcatheter aortic valve procedures (transfemoral, transapical or transaortic), patients developing endocarditis during index admission/surgery due to endocarditis or patients transferred to neurological rehabilitation unit due to perioperative stroke, Fig. 1.

### 2.2 Intervention

The intervention was initiated prior to discharge and included a clinical examination comprising a focused chest ultrasound (to assess potential pleural or pericardial effusion), ECG-screening for rhythm disorders, a medical evaluation, a frailty test and patient education. Based on the included elements, a risk assessment was performed, Fig. 2.

Follow-up after discharge was planned according to the risk assessment and performed by specialised nurses with the possibility of consulting cardiologists and heart surgeons, when clinically indicated.

Patients considered being at high risk of readmission were seen more frequently in the outpatient clinic compared with patients being in intermediate or low risk of readmission, Fig. 2. The elements of the intervention have been described more thoroughly in the main outcome paper [1].

### 2.3 The historical control group

Patient in the historical control group received a short, unstructured telephone consultation within the first week after discharge from either the surgical ward or a local hospital.

All patients (in both intervention and control group), were referred back to general practitioner for removal of stitches. They furthermore underwent an echocardiography according to European guidelines [2] 4–6 weeks after surgery and were afterwards offered participation in cardiac rehabilitation.

### 2.4 Readmission and mortality

In this dataset, we defined a readmission as a new admission (with an overnight stay) occurring more than 24 hours after discharge and within 180 days after discharge. Readmissions included were unplanned readmissions due to cardiac causes or causes related to the surgery. Registration of readmissions, including causes of readmission, was based on electronic medical records. Causes of readmission were grouped into *pericardial effusion*, *pleural effusion*, *atrial fibrillation/flutter*, *heart failure*, *infections* (pneumonia, endocarditis, sternal infections and unspecified infections), *cardiac symptoms without other specific cause* (angina pectoris, dyspnea, vertigo or syncope) and *others presumed related to the surgery* (e.g., anemia, dysregulation of anti-coagulation therapy, new pacemaker implantation, stroke, acute coronary syndrome, re-operation, wound problems, gastro-intestinal bleeding, medical problems (not anti-coagulation) and ventricular tachycardia).

Data on all-cause mortality were obtained from electronic medical records.

### 2.5 Statistical analyses

Data were analysed using SPSS 24 (IBM Corp, Armonk, NY). Continuous data (days readmitted) were presented as mean and standard deviation (SD) and compared using two-sample *t*-test. Categorical data (readmissions, causes of readmission and mortality) were presented as number of patients and percentages and compared using χ^2^ (categorical data), Table 1, Table 2

The effect of the intervention was analysed using univariable Cox proportional hazard regression model in both the overall population and a propensity matched group [1]. The propensity score matching was performed with a 1:2 matching without replacement. We used the nearest-neighbour approach and a caliper width of 0.2 SD, as previously suggested [3], [4]. The propensity model included: sex, age, acute/unplanned surgery, primary diagnosis, type of surgery, concomitant coronary artery bypass surgery, obstructive or restrictive lung disease, New York Heart Association class (NYHA), EuroScore II (logistic), estimated glomerular filtration rate, permanent pacemaker prior to surgery, atrial fibrillation and body mass index (BMI) [1].

In addition to the univariable model, multivariable Cox proportional hazard analyses were performed. All analyses of primary outcome were conducted according to the intention-to-treat principle. The propensity matched population and results of the multivariable Cox proportional hazard analyses are presented elsewhere [1].

We performed sensitivity-analyses to investigate other potential differences among the intervention and historical control group, including differences in number of unplanned/planned readmissions, all-cause readmission, all-cause mortality, mean length of stay per readmission, number of readmissions per patient and total number of readmissions, Table 2.
